# Treatment of long-term endophthalmitis developing after intraocular lens implantation in children: a retrospective study

**DOI:** 10.1186/s12886-022-02519-3

**Published:** 2022-07-12

**Authors:** Chen Zou, Ting Zhang, Xin Wang, Hong Zhuang, Rui Jiang

**Affiliations:** Department of Ophthalmology, Eye and ENT Hospital of Fudan University, 83# Fenyang Road, Shanghai, China

**Keywords:** Long-term endophthalmitis, IOL implantation, Vitrectomy, Retrospective, Risk factor

## Abstract

**Background:**

To discussed the risk factor and the management of vitrectomy for long-term endophthalmitis developing after intraocular lens (IOL) implantation in children.

**Methods:**

We retrospectively investigated the clinical characteristics and surgical outcomes of long-term endophthalmitis developing after IOL implantation in children.

**Results:**

Four eyes of four children were included in the study. The mean time to endophthalmitis development after IOL implantation was 3.0 ± 0.8 years. The corneal or scleral sutures may have been caused the infection. All 4 patients underwent vitrectomy and received intravitreal antibiotics with or without IOL removal. At the last follow-up, the outcomes were satisfactory; the fundus was clear, the retina remained attached, the visual acuity improved, and there were no severe complications.

**Conclusions:**

The use of scleral sutures and the exposure of conceal sutures may induce the onset of long-term endophthalmitis after IOL implantation. Complete vitrectomy and appropriate use of antibiotics are effective in the treatment of long-term endophthalmitis developing after IOL implantation in children.

## Background

Lens aspiration followed by primary or secondary intraocular lens (IOL) implantation is common in treating lens diseases, including lens dislocation in Marfan syndrome (MFS), congenital cataracts, or ocular trauma in children. Although the technology of IOL implantation or fixation has advanced rapidly in decades, endophthalmitis is still the most serious complication of IOL implantation that could occur within days to years after surgery and often triggers severe visual impairment or even eye loss. Although post cataract endophthalmitis has been widely reported, with an incidence ranging from 0.012 to 1.3% in a large meta-analysis [1], endophthalmitis, especially long-term endophthalmitis, occurring after lens aspiration and IOL implantation in pediatric patients, has rarely been reported. Therefore, the consensus on the surgical treatment of long-term endophthalmitis in children has not been improved.

Vitreous surgery is critical in managing most infectious endophthalmitis cases, as it can reduce the number of organisms, immune cells, and soluble mediators and enhance the diffusion of antibiotics. However, vitreous surgery in children with endophthalmitis is a huge challenge, including difficulty in inducing posterior vitreous detachment, difficulty in complete vitrectomy, recurrence of postoperative infection, and requirement for anesthesia. In this study, in order to share our experience about the risk factor and the management of vitrectomy for long-term endophthalmitis developing after IOL implantation in children, we retrospectively reviewed the clinical characteristics and surgical outcomes of four children who developed long-term post-implantation endophthalmitis. These results may help to improve the consensus on the surgical treatment of long-term endophthalmitis in pediatric patients.

## Methods

 This study adhered to the Declaration of Helsinki and was approved by the Institutional Review Board of the Eye and Ear, Nose, and Throat Hospital of Fudan University, Shanghai, China. Informed consent about the study were obtained from the parents of all the patients. We retrospectively reviewed the clinical records of patients with endophthalmitis with a history of IOL implantation at the Department of Ophthalmology from 2020 to 2021. Endophthalmitis was diagnosed based on ocular symptoms, evidence of infection in the anterior chamber, ultrasound data, and history of IOL implantation.

All patients underwent vitrectomy. The surgery was performed with the vitrectomy system (Bausch & Lomb, Stellaris, USA), operating microscope (Carl Zeiss, OPMI VISU 200, Germany) and the non-contact wide-angle imaging system (Biom, Germany). All the patients received general anesthesia. The anterior chamber was cleared firstly. The vitrectomy instrument was used to clear the anterior chamber under anterior chamber perfusion if necessary. After cleaning the anterior chamber, infusion cannula was implanted 3 mm posterior to the inferior temporal limbus, and the perfusion was opened on the premise that the perfusion cannula could be seen clearly through the pupil. Then, the incision for the vitrectomy instrument and endoilluminator were made in the usual position. The corneal epithelium was removed and viscoelastic was used on the surface of cornea if the corneal edema hinders the visualization during vitrectomy. The anterior, central, and peripheral vitreous bodies were excised successively. IOL was removed if necessary, such as IOL opaque or ciliary membrane formation. Intravitreal injection of antibiotics (Norvancomycin 0.8 mg + Ceftazidime 2.25 mg, the same below) was made at the end of the surgery. Personalized surgical details of each patient are described below. Intravenous ceftazidime 50 mg/kg qd, topical tobramycin, moxifloxacin, atropine and prednisolone were used postoperatively.

We recorded demographic data, duration of IOL implantation, cause of infection, best-corrected logMAR visual acuity (BCVA; Snellen visual acuity before and after vitrectomy [finger count, FC/1 ft = 1/100; hand motion, HM/1 ft = 1/1,000; and light perception, LP = 1/10,000) before and after vitrectomy, surgical features, and bacterial culture results. Data are presented as the mean ± standard deviation. The Mann–Whitney U test was used to compare the clinical variables. Statistical significance was set at *p* < 0.05. All statistical analyses were performed using the SPSS software for Windows (ver. 17.0; SPSS Inc, Chicago, IL, USA).

## Results

### Baseline findings

We enrolled 4 pediatric patients (one male and three females) with endophthalmitis. All the patients had a history of MFS and were treated with lens aspiration and primary or secondary IOL implantation. Double-loop posterior chamber IOLs were used in the previous surgery in all the patients. The demographic data are summarized in Table 1. The average age at endophthalmitis development was 10.25 ± 1.7 years (range: 8–12 years). The average time to the onset of endophthalmitis after IOL implantation was 3.0 ± 0.8 years (range: 2–4 years). During IOL implantation, two patients underwent IOL scleral fixation and two other patients underwent capsular tension ring (CTR) implantation or trans scleral modified CTR (MCTR) fixation.

**Table 1:** Demographic data and Preoperative clinical characteristics

No.	Age (y)	Sex	Onset period (y)	IOL fixation	Period before vitrectomy	Infection inducement	preoperative BCVA	Cornea	Anterior chamber
1	11	female	3	scleral	6	sclera suture	HM	edema	purulent exudation, cell++++
2	8	female	2	CTR	3	cornea suture	LP	ulceration around suture	purulent exudation, cell++++
3	10	male	4	scleral	17	sclera suture	LP	edema	cell++
4	12	female	3	MCTR	3	cornea suture	LP	edema, suture was removed	Hypopyon 1 mm cell++++

*IOL* intraocular lens, *CTR* capsular tension ring, *MCTR* modified capsular tension ring, *HM *Hand movement, *LP* light perception

### Preoperative clinical characteristics

The time between vitrectomy and the onset of ocular symptoms ranged from 3 to 17 days. The BCVA before vitrectomy ranged from LP to HM. The condition of the cornea and anterior chamber, ultrasound data, and other clinical findings are summarized in Table 1. Suturing of the corneal limbus or sclera may have caused the infections. The corneal limbus sutures of patients #2 and #4 were exposed, and an unremoved suture of the corneal limbus was evident in the fellow eye of patient #4. The two other patients previously underwent transscleral IOL fixation with sutures, and these sutures were not exposed to the conjunctiva.

### Treatments and outcomes

All patients underwent vitrectomy at our hospital. Patient #1 underwent a single vitrectomy; however, the three other patients underwent two vitrectomies prior to infection control. Antibiotics were injected intravitreally intraoperatively, and all patients received intravenous antibiotics after vitrectomy. At the last follow-up in all patients, the anterior segment and fundus were clear and the retina remained attached. Post operative BCVA ranged from FC/1 ft to 20/50. The details are presented in Table 2.

**Table 2:** Surgical characters and outcomes

No.	Ciliary membrane	Times of vitrectomy	IOL	Silicone oil tamponade	last follow up time	BCVA at last follow up	IOP (mmHg)
1	Yes	1	removed	Yes	7d	5/100	13
2	No	2	removed	Yes	11 m	15/100	7.2
3	Yes	2	removed	Yes	4 m	FC/1ft	4
4	No	2	retained	No	4 m	20/50	12

*IOP* intraocular pressure

**Fig. 1:**
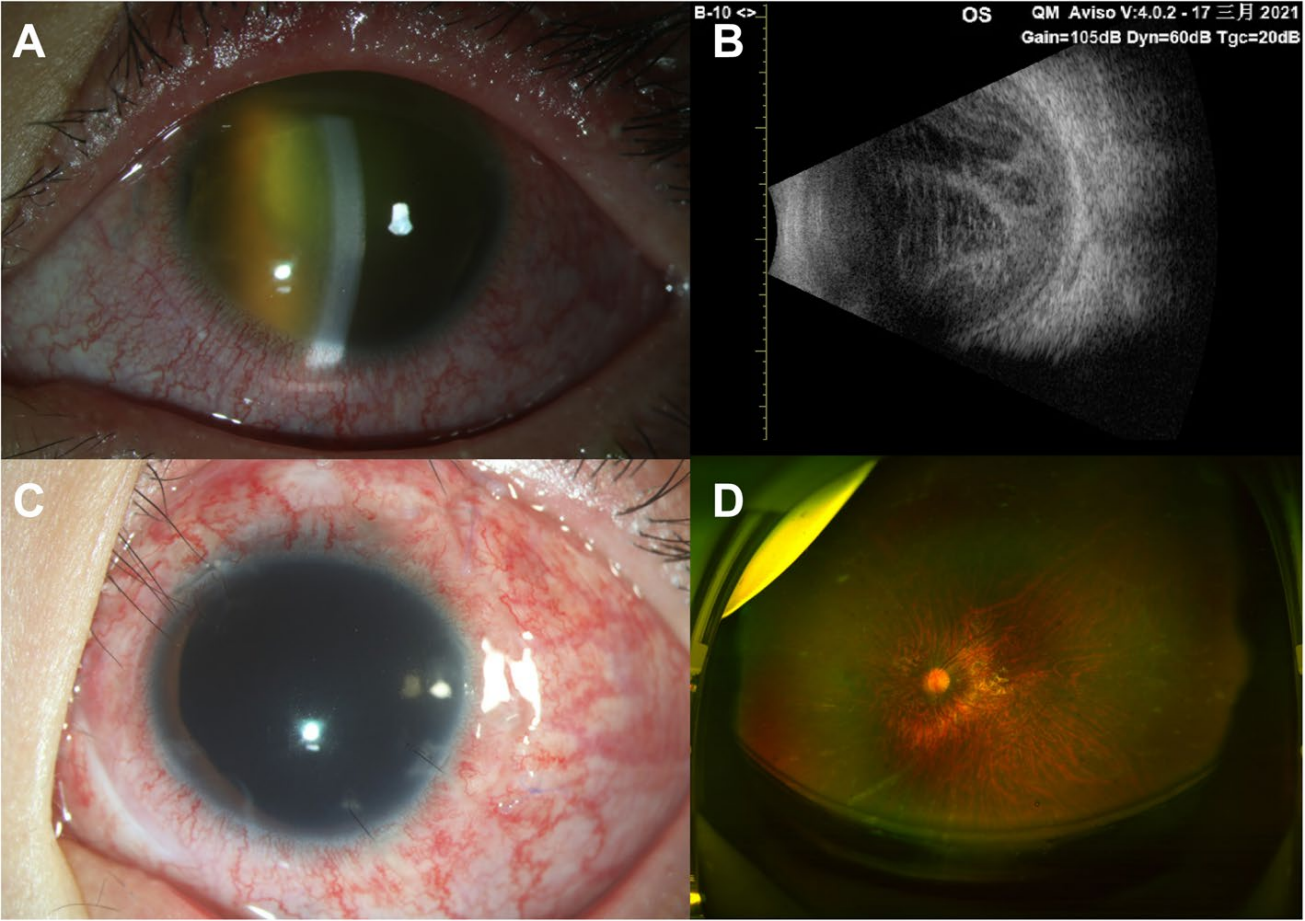
For patient #1. **A** anterior segment photography before vitrectomy. **B** B-ultrasound before vitrectomy. **C** anterior segment photography at the latest follow-up. **D** Ultra-wide angle fundus photography at the latest follow-up

Patient #1 underwent complete and early vitrectomy for endophthalmitis (CEVE, described by Dib et al. [2]). The IOL was removed, and antibiotics and a silicone oil tamponade were injected because a ciliary membrane was apparent. The peripheral vitreous and ciliary membranes were excised maximally. Two days later, the infection was controlled, and the fundus was faintly apparent (Fig. 1). The bacterial culture was negative. Seven days after vitrectomy, which was also the last follow-up, the BCVA was 5/100, and the intraocular pressure (IOP) was normal.

**Fig. 2:**
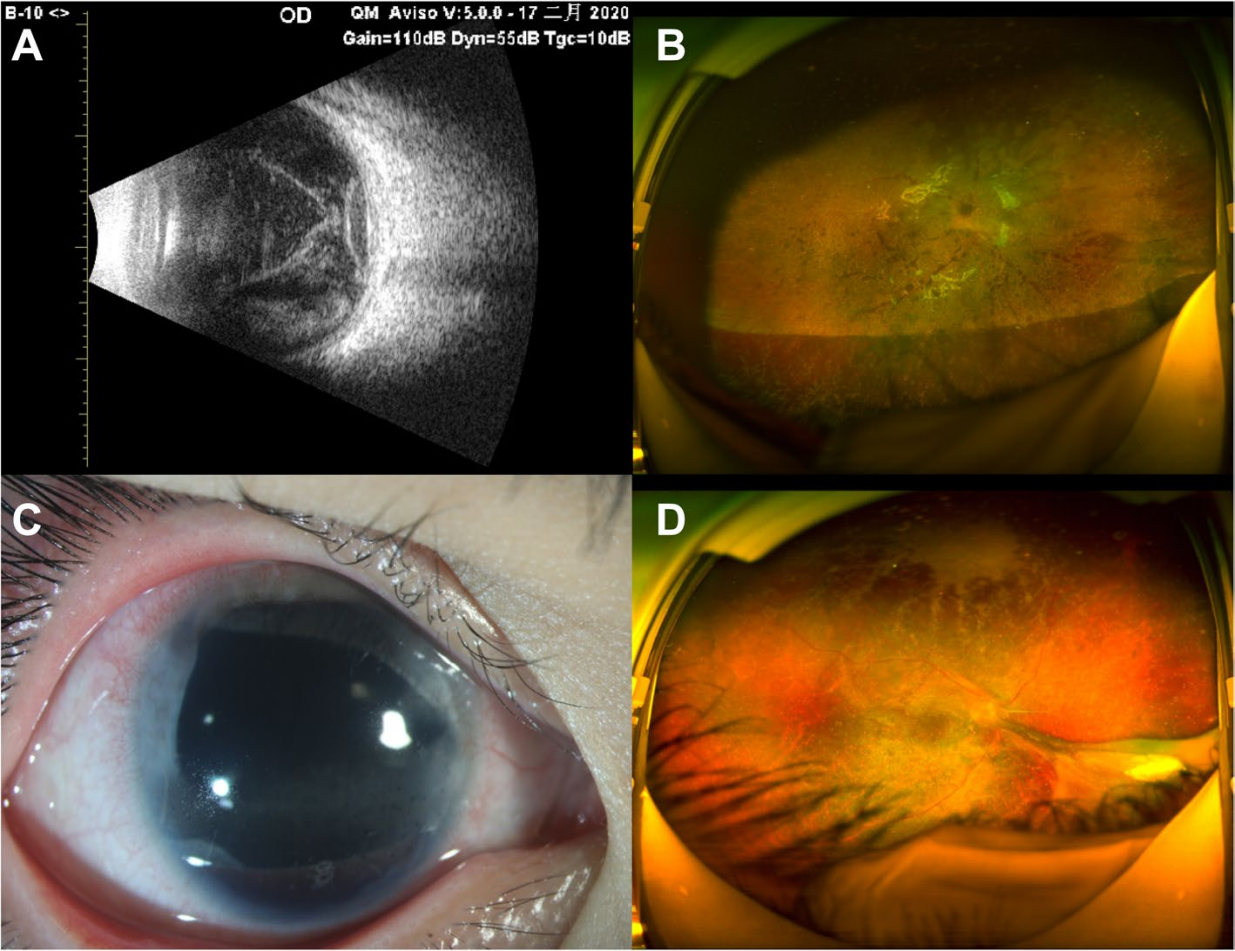
For patient #2. **A** B-ultrasound 2 days after the first vitrectomy. **B** Ultra-wide angle fundus photography 2 months after the second vitrectomy. **C** anterior segment photograph y 10 months after the second vitrectomy. **D** Ultra-wide angle fundus photography at the latest follow-up

Patient #2 underwent emergency vitrectomy and intravitreal antibiotics; however, no gas or silicone oil tamponade was injected. No ciliary body membrane was apparent during surgery. Three days later, complete vitrectomy was repeated because infection control was unsatisfactory. The IOL and CTR were removed, and silicone oil tamponade was applied. The peripheral vitreous and ciliary body membranes were excised maximally. Seven days after the second vitrectomy, the infection was controlled, and the fundus was faintly apparent (Fig. 2). Cultures revealed *Staphylococcus hominis* infection of both the vitreous sample and the corneal suture. At the last follow-up (11 months later), BCVA was 15/100, and the IOP was 7.2 mmHg. The anterior segment and fundus were clear; however, the nasal retina exhibited traction.

**Fig. 3:**
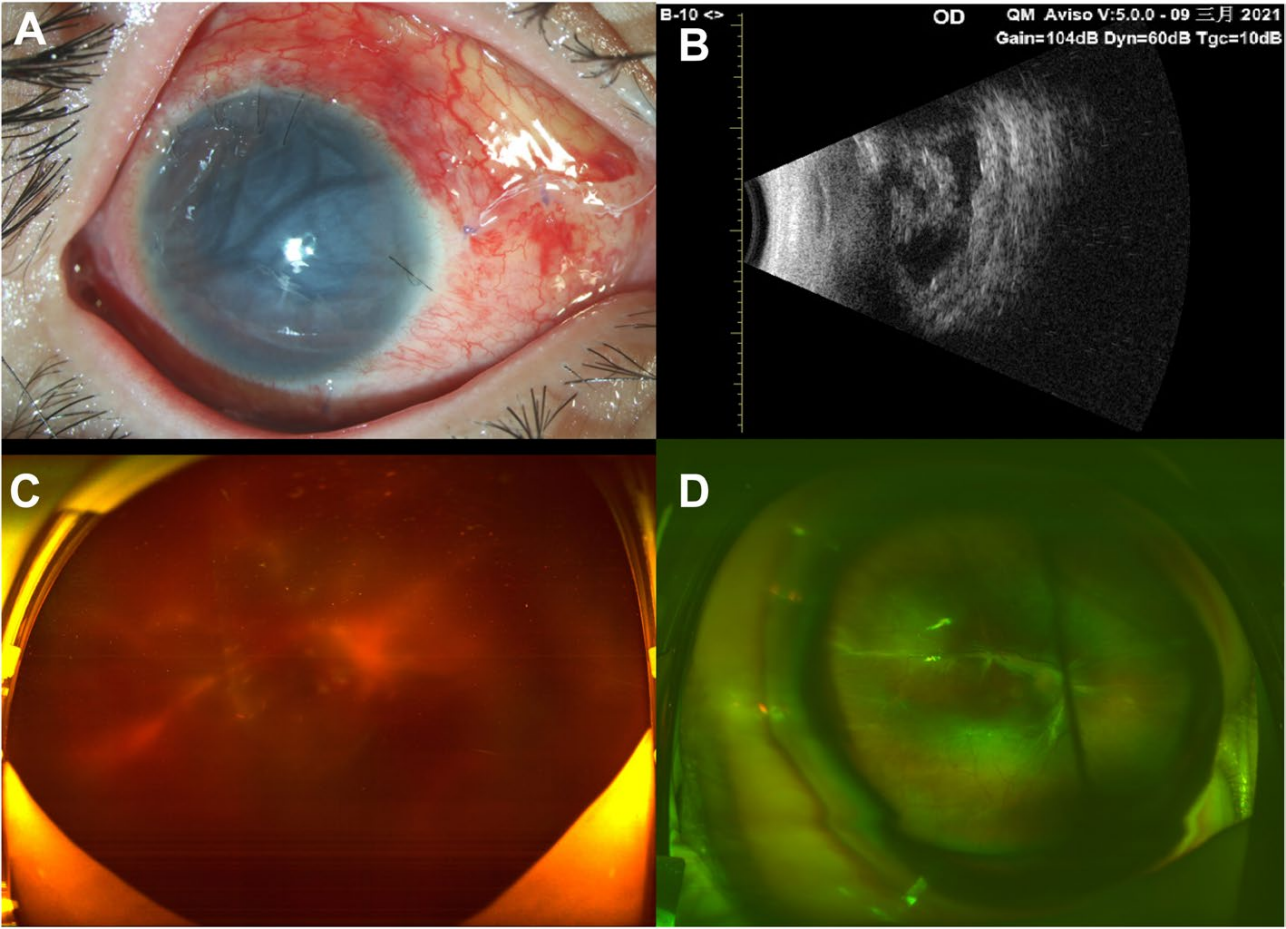
For patient #3. **A** anterior segment photograph 3 days after the first vitrectomy. **B** B-ultrasound 3 days after the first vitrectomy. **C** Ultra-wide angle fundus photography 8 days after the second vitrectomy. **D** Ultra-wide angle fundus photography at the latest follow-up

Patient #3 initially underwent vitrectomy, the IOL was removed, and intravitreal antibiotics were injected without gas or silicone oil tamponade. Four days later, the patient underwent a repeat complete vitrectomy because the infection was not well controlled. A ciliary body membrane was found and maximally excised, together with the peripheral vitreous membrane. Intravitreal antibiotics were injected, and silicone oil tamponade was applied. Two days later, the infection was controlled, and the fundus was faintly visible (Fig. 3). Culture revealed *Staphylococcus* infection of the vitreous membrane. Four months later, the BCVA was FC/1 ft, and the IOP was 4 mmHg. The fundus was clear. However, traction and extensive choroidal detachment were apparent and were associated with poor visual acuity and low IOP.

**Fig. 4:**
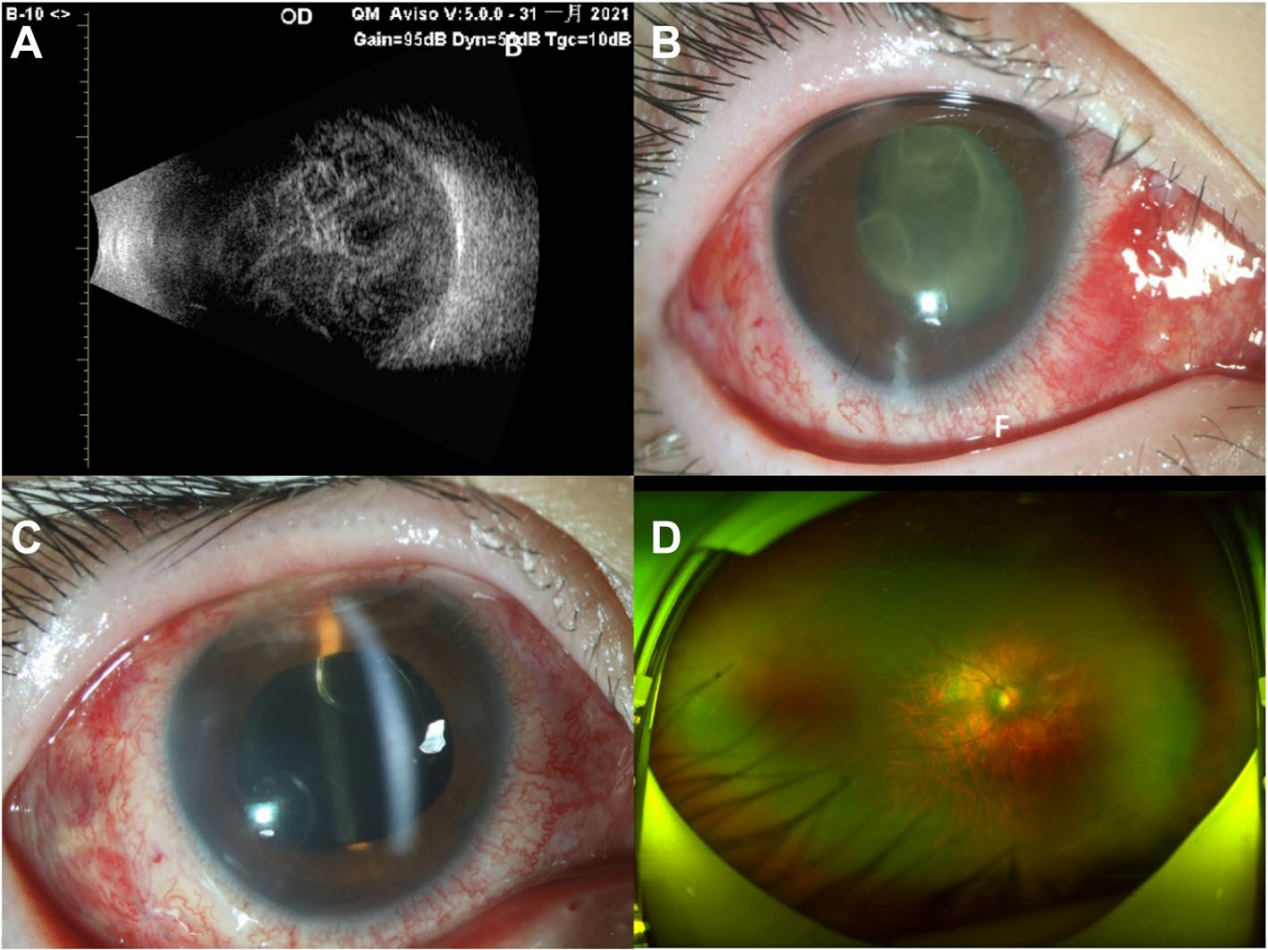
For patient #4. **A** B-ultrasound before the first vitrectomy. **B** anterior segment photograph 3 days after the first vitrectomy. **C** anterior segment photograph 2 days after the second vitrectomy. **D** Ultra-wide angle fundus photography at the latest follow-up

Patient #4 first underwent corneal suture removal after the suture was exposed following endophthalmitis onset (observed in a local hospital). The patient presented to our emergency room at night and received emergency intravitreal antibiotics; no vitrectomy surgeon was on call. The next day, she underwent vitrectomy, intravitreal antibiotic injection, and removal of the corneal limbus suture from the fellow eye. Two days later, she received additional intravitreal antibiotics because the infection was not well controlled. However, the infection remained uncontrolled for the next 3 days, at which time she underwent a second complete vitrectomy, capsulectomy, and intravitreal antibiotic injection. Neither gas nor silicone oil was injected. Four days after the second vitrectomy, the infection was better controlled, and the fundus was faintly observed (Fig. 4); the microbial culture was negative. The last follow-up was 4 months later; the BCVA was 20/50, and the fundus was clear.

## Discussion

In the current study, we reported four cases of long-term endophthalmitis that developed after IOL implantation in children and retrospectively evaluated their surgical management and outcomes. Endophthalmitis is a severe complication that may develop days to years after lens aspiration and IOL implantation, causing irreversible blindness, eye pain, and the need for eye enucleation. The risk factors for endophthalmitis after lensectomy and IOL implantation include lacrimal duct obstruction, blepharitis, use of contact lenses, long surgical time, posterior capsular rupture, scleral suture fixation, corneal suturing, and the polypropylene loop of the IOL [3]. However, studies on endophthalmitis that develops after IOL implantation in children are rare. Asadi et al. [4] reported long-term surgical complications in 25 eyes of 23 children who underwent implantation of transscleral fixated posterior chamber IOLs, including six eyes of four patients with lens dislocation caused by MFS. Endophthalmitis developed in one patient with MFS approximately 3 years after IOL implantation. Kristianslund et al. [5] reported 132 eyes (92 patients) that underwent MCTR implantation with scleral suturing. One eye (0.8%) developed late endophthalmitis, possibly related to suture exposure. Kanigowska et al. [6] reported that the endophthalmitis rate after lensectomy, vitrectomy, and IOL implantation with scleral fixation was 0.8% (1/116) in children exhibiting lens dislocation. Recently, Chen et al. [7] reported the surgical outcomes of MCTR and IOL implantation in patients with MFS and ectopia lentis. A total of 174 eyes with MFS underwent surgery. One eye of a patient (0.57%) in their cohort developed endophthalmitis 19 months after the corneal suture was removed. Although the above studies reported sporadic cases of endophthalmitis after IOL implantation in children, study focusing on the surgical treatment of long-term endophthalmitis after IOL implantation in children is rare. No consensus has been reached regarding the management of vitreous surgery for long-term endophthalmitis following IOL implantation in children. Therefore, we reviewed relevant cases and surgical outcomes to share our experience.

In the current study, all four children had a history of lens dislocation caused by MFS, an uncommon autosomal-dominant pleiotropic connective tissue disease, known to affect many systems, including the ophthalmological, musculoskeletal, and pulmonary systems. Ocular conditions may be the initial presenting symptoms in patients in whom cardiovascular symptoms have not yet developed. These symptoms include lens dislocation, myopia, glaucoma, cataracts, and retinal detachment [8]. Lensectomy followed by IOL implantation effectively treats lens dislocation that characterizes MFS. However, whether MFS is a risk factor for long-term endophthalmitis after IOL implantation remains unknown. The possible causes of long-term infection are the existence of corneal or scleral sutures, which can also be seen in other lens surgeries in children, such as surgeries for congenital cataract or ocular trauma. Further large sample multiple-factor analysis studies are necessary to confirm the role of MFS in long-term endophthalmitis development after lens aspiration and IOL implantation in children.

Corneal sutures were an important possible cause of long-term endophthalmitis in our study. Two of the four patients had a history of corneal suture exposure, which may cause microorganisms on the surface to penetrate the cornea, causing endophthalmitis even several years after the previous surgery. This reveals the importance of removing corneal sutures appropriately after cataract surgery or IOL implantation, although general anesthesia must be induced prior to suture removal in children. The direction of removal is also important. The suture should not contact the surface of the cornea as this may introduce microorganisms into the interior of the eye.

Another reason for the long-term onset of endophthalmitis is the scleral suture. The exposure of suture knots has been reported as a long-term complication of scleral IOL fixation [9]; however, in our cases of endophthalmitis developing after IOL fixation, the sutures were not exposed on the surface of the cornea or conjunctiva. However, microorganisms may access the internal eye via scleral sutures associated with the near-invisible gaps on the eye surface. Although scleral flap placement reduces the risk of suture exposure, suture ends can penetrate partial-thickness scleral flaps and conjunctiva in the long term [4]. Therefore, suture ends should not be exposed. This can be prevented by leaving the suture ends long, rotating the knots into the sclera, or tying the knots at the depth of the partial-thickness scleral incision [10]. In 2007, Scharioth introduced transscleral fixation of a three-piece IOL using intrascleral tunnels [11]. Recently, Yamane et al. [12] reported a new flanged IOL fixation technique to achieve good IOL fixation with firm haptic fixation. These suture-free techniques prevent suture-related complications.

In our study, all patients underwent complete vitrectomy, although three of the four patients required two vitrectomies prior to complete control of the infections. The utility of complete and core vitrectomy as treatments for post cataract endophthalmitis remains controversial. In the Endophthalmitis Vitrectomy Study (EVS) conducted in the 1990s, the question was raised whether pars plana vitrectomy (PPV) was superior to vitreous tap/biopsy (VTB) in conjunction with broad-spectrum intravitreal antibiotics. It was found that the three-port PPV and VTB were equivalent in eyes with vision better than LP. In LP eyes, vitrectomy provided significantly better visual results [13]. However, given the advances in vitrectomy techniques in recent decades, experts have questioned whether the EVS findings remain applicable. The principal limitation of the EVS was that all vitrectomies were of the core type, and removal of cortical vitreous purulence on the retinal surface was explicitly discouraged to avoid iatrogenic retinal tears. Dib et al. [2] recommended complete vitrectomy for all eyes with infections that obscured the fundus, including posterior vitreous detachment if required. The main advantage of complete vitrectomy compared with core vitrectomy is the removal of purulence from the cortical vitreous and retinal surface, which limits retinal injury caused by endophthalmitis. In our clinical practice, the type of vitrectomy used depends on many factors, including the extent of infection, transparency of the refractive medium, presence/absence of a ciliary membrane, and the surgeon’s ability. In patient #1, a ciliary membrane was formed prior to vitrectomy; thus, we performed an initial CEVE and IOL removal. The outcome 7 days after vitrectomy was satisfactory; however, the patient was lost to follow-up. The three other patients underwent initial incomplete vitrectomies; however, the extent was wider than that of core vitrectomy and VTB. Unfortunately, the infections were not controlled, and all three patients underwent a second (complete) vitrectomy.

The IOL was removed from three of the four patients. Capsulectomy was performed in patient #4 (whose IOL was retained). These factors promote intraocular fluid circulation and control infection and inflammation. The necessary for IOL removal remains controversial because of increased surgical difficulty and possible complications, including retinal detachment, hypotony, corneal edema, endothelial decompensation, macular edema, and vitreous or subretinal hemorrhage [14]. Zhang et al. [15] retrospectively evaluated surgical efficacy and determined when IOL removal was indicated during vitrectomy to treat endophthalmitis. It was concluded that IOLs should not be removed. However, it remains unclear whether IOL removal is necessary when endophthalmitis develops long after IOL implantation in children’s patients, and further research is needed. We removed IOLs that were opaque and associated with severe anterior vitreous symptoms, such as ciliary membrane formation.

This research also had some shortcomings, including the small sample, the retrospective nature, the lack of a control group. Moreover, one patient lost follow-up early on. Further controlled clinical study is necessary.

## Conclusions

In summary, we retrospectively reviewed long-term endophthalmitis after IOL implantation in children. All four patients underwent complete vitrectomy and intravitreal antibiotic injection, with or without IOL removal, and the outcomes were satisfactory. The use of scleral sutures and the exposure of conceal sutures may induce the onset of long-term endophthalmitis after IOL implantation. Removal of the corneal sutures postoperatively is suggested.

## Data Availability

All data generated or analysed during this study are included in this published article.

## Abbreviations

IOL: Intraocular lens
MFS: Marfan syndrome
BCVA: Best-corrected visual acuity
FC: Finger count
HM: Hand motion
LP: Light perception
CTR: Capsular tension ring
MCTR: Modified capsular tension ring
CEVE: Complete and early vitrectomy for endophthalmitis
IOP: Intraocular pressure
EVS: Endophthalmitis Vitrectomy Study
PPV: Pars plana vitrectomy
VTB: Vitreous tap/biopsy
